# High-resolution surface electromyographic activities of facial muscles during the six basic emotional expressions in healthy adults: a prospective observational study

**DOI:** 10.1038/s41598-023-45779-9

**Published:** 2023-11-06

**Authors:** Orlando Guntinas-Lichius, Vanessa Trentzsch, Nadiya Mueller, Martin Heinrich, Anna-Maria Kuttenreich, Christian Dobel, Gerd Fabian Volk, Roland Graßme, Christoph Anders

**Affiliations:** 1 Department of Otorhinolaryngology, Jena University Hospital, Friedrich-Schiller-University Jena, Am Klinikum 1, 07747 Jena, Germany; 2 Facial-Nerve-Center Jena, Jena University Hospital, Jena, Germany; 3 Center for Rare Diseases, Jena University Hospital, Jena, Germany; 4 Division Motor Research, Pathophysiology and Biomechanics, Department of Trauma, Hand and Reconstructive Surgery, Jena University Hospital, Friedrich-Schiller-University Jena, Jena, Germany; 5 Department of Prevention, Biomechanics, German Social Accident Insurance Institution for the Foodstuffs and Catering Industry, Erfurt, Germany

**Keywords:** Human behaviour, Neurological disorders

## Abstract

High-resolution facial surface electromyography (HR-sEMG) is suited to discriminate between different facial movements. Whether HR-sEMG also allows a discrimination among the six basic emotions of facial expression is unclear. 36 healthy participants (53% female, 18–67 years) were included for four sessions. Electromyograms were recorded from both sides of the face using a muscle-position oriented electrode application (Fridlund scheme) and by a landmark-oriented, muscle unrelated symmetrical electrode arrangement (Kuramoto scheme) simultaneously on the face. In each session, participants expressed the six basic emotions in response to standardized facial images expressing the corresponding emotions. This was repeated once on the same day. Both sessions were repeated two weeks later to assess repetition effects. HR-sEMG characteristics showed systematic regional distribution patterns of emotional muscle activation for both schemes with very low interindividual variability. Statistical discrimination between the different HR-sEMG patterns was good for both schemes for most but not all basic emotions (ranging from p > 0.05 to mostly p < 0.001) when using HR-sEMG of the entire face. When using information only from the lower face, the Kuramoto scheme allowed a more reliable discrimination of all six emotions (all p < 0.001). A landmark-oriented HR-sEMG recording allows specific discrimination of facial muscle activity patterns during basic emotional expressions.

## Introduction

It is assumed that a person’s emotional state can be readily inferred from his or her facial movement patterns, typically called emotional expressions^1^. Emotional expressions are assumed to inform others about the counterparts' affective states^2,3^. Facial expressions are a means of communication that are more rapid than language, with which people can quickly infer state of mind of their companions^4^. Hence, facial expression are a powerful tool of non-verbal communication in social coordination and therefore of major interest in psychological research. Facial surface electromyography (sEMG) is the standard tool to f measure facial expressions of emotions in a laboratory context of psychological and physiological experiments^1,5^. Traditional science defines six basic prototypical facial expressions when analyzing emotions: anger, disgust, fear, happiness, sadness, and surprise^6^. Basic emotion theory proposes that the basic emotions are manifested through organized and recurrent movement patterns of behavior, which can be read mainly from the face.

Typically, anatomically defined facial muscles are recorded by sEMG in psychophysical experiments to differentiate between emotions of positive and negative valence. Main examples are the zygomaticus major (important for smiling) and the corrugator muscles (involved in frowning), which have been extensively investigated to differentiate positive and negative valence^7,8^. In contrast to the physiological method, the Facial Action Coding System (FACS) is a standard method to visually assess facial expressions in order to identify discrete facial muscle movements, called Action Units (AUs), i.e. without use of EMG. AUs are activated during different emotional facial expressions^9^. The supposed anatomical and muscular origin for each AU is the activity of one to three facial muscles^10^. Originally, the assignment of the AUs to individual mimic muscles was based on EMG analyses^9^. There is, however, an ongoing debate as to whether reading from those muscles alone can suffice for detection of specific emotional states or if groups of muscles should also be taken into account^3,11^. Similar configurations of facial movements variably express instances of more than one emotion category pointing towards a large overlap^1^. It is thus, an open question, which electrode configurations allow to discriminate the basic emotions.

Hence, multi-channel high-resolution facial surface electromyography (HR-sEMG) might be a more precise methodological approach to measure basic emotional expressions than only EMG recording from a small set (of only one to three) facial muscles. Most functional (non-emotional) facial tasks lead to a complex sEMG activation of several or even almost all facial muscles stressing the need and value of multi-channel recordings^12–14^. Recently, we showed that a HR-sEMG recording scheme by Kuramoto et al.^15^ covering the complete face in an EEG-like landmark-oriented and symmetrical arrangement seems to result in more distinction and specific facial muscle activity patterns during various functional (non-emotional) facial expression tasks than the most popular scheme by Fridlund and Cacioppo^16^ with sEMG recordings from ten specific mimic and one masticatory muscles, i.e. the user needs specific knowledge of the facial muscle topography^17^. In addition, the scheme by Kuramoto et al. showed a higher inter-session reliability when repeating the measurements in the same probands at several days^18^.

Therefore, as a next step we wanted to study the simultaneous application of both schemes during the six basic emotions. We used the same methodological setting for HR-sEMG to measure basic emotional expressions in healthy probands. The following hypotheses were tested: (1) the Kuramoto scheme is more reliable than the Fridlund scheme for the differentiation of different emotional expressions; (2) the Kuramoto scheme shows a better re-test reliability than the Fridlund scheme for the analysis of emotional expressions and the reliability is different for different emotional expressions; (3) In general, it is doubtful whether complex movement profiles of muscle chains can be imaged with few EMG electrodes. In general, it is doubtful whether complex movement profiles of muscle chains require electrodes covering the entire face, or, if emotional expressions can also be distinguished with high certainty by recordings of only parts of the face like the upper and lower part.

## Materials and methods

### Healthy participants

**Figure 1:**
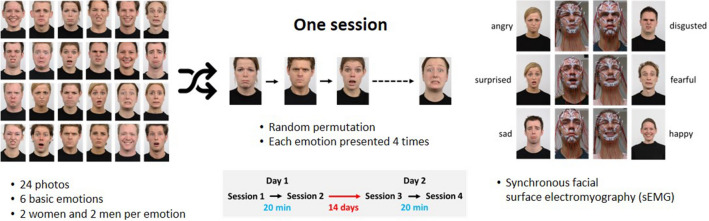
Experimental setting. Four sessions were performed by each participant. Each session consisted of the permuted presentation of 24 photos showing the six basic emotions presented by two males and two females, respectively. The surface electromyography was performed simultaneously. The first two sessions were performed in one day with a time lag of 20 min. The interval between session 2 and session 3 was about 14 days. Session 3 and 4 were again performed in one day, again with a time lag of 20 min.

Nineteen (19) women and 17 men were included (age range: 18–67 years) were included. As inclusion criterion, the participants had to be healthy. Subjects with a history of any neurological disease, or an active neurological disease as well as a history of facial surgery, were excluded. Only beardless men were recruited. Only beardless men were recruited. Results on the functional measurements were published recently^17,18^. All experimental procedures with human subjects followed the institutional research committee's ethical standards and the 1964 Helsinki Declaration and its later amendments. The ethics committee of the Jena University Hospital approved the study (No. 2019–1539). All participants gave written informed consent to participate in the study. Informed consent has also been obtained to publish the facial images in an online open-access publication shown in Fig. 1.

### Standardization of the execution of the six emotional expressions

The participants were instructed about the sequence of the examination. The instructions for the emotional expressions presented by a video tutorial were explained. Details of the video tutorial are presented elsewhere^19^. The participants sat in relaxed upright position in front of a computer screen and followed the self-explanatory video tutorial. Participants performed first eleven functional facial exercises (for instance, eye closure or wrinkling of the forehead). The analyses of these tasks were published recently^17,18^. The subject of the present experiment is the following part of the investigation: A permuted sequence of 24 permuted photos featuring different male and female faces expressing the six basic emotions (fear, surprise, joy, sadness, anger, disgust) were presented^5,20^. Within this sequence, each emotion was present four times. Each emotion was expressed by two male and two female faces. Participants were instructed to imitate the facial expressions as best they could without explicit information which emotion was intended. In total, four recordings (= sessions) were performed **(**Fig. 1). On day 1, two sessions (session 1 and session 2) were performed with a time lag of about 20 min. Sessions 3 and 4 were performed at day 2. Day 2 was 14 days later with the same time lag of 20 min between the sessions^18^. Hence, it was possible to calculate the intra-session reliability, the intra-day reliability, and the between-day reliability of HR-sEMG for individual movement patterns.

### High-resolution facial surface electromyography (HR-sEMG) registration

The HR-sEMG protocol was published recently^17,18^. Bilateral facial electromyograms were recorded. Prior to data collection, all participants cleaned their face thoroughly with non- refatting medical soap to minimize skin impedance. The equipment consisted of reusable surface electrodes (Ag–Ag–Cl discs, diameter: 4 mm, DESS052606, GVB-geliMED, Bad Segeberg, Germany), reference electrodes (H93SG, Kendall, Germany, bilaterally attached to the mastoid bone), sEMG amplifiers (ToEM16G, gain 100, frequency range 10–1,861 Hz, DeMeTec, Langgöns, Germany), and analogue to digital conversion (Tom, resolution: 5,96nV/Bit, sampling rate: 4096/s, cutoff frequency: 2048 Hz, DeMeTec, Langgöns, Germany). Data were sampled (ATISArec, GJB Stentechnik, Ilmenau, Germany) and stored on hard disk for further analysis. Two electrode arrangements were applied simultaneously: The classical scheme developed by Fridlund and Cacioppo (shortly named “Fridlund” in the following)^16^ was used as well as the recently published schema by Kuramoto et al. (shortly “Kuramoto”)^15^. To ensure reliable electrode positioning, application of the two schemes were done by two well experienced examiners (NM and VT), adhering to a strictly defined application mode and with the additional use of template foils. EMG amplitudes were quantified as mean root mean square (RMS) values during the steady state contraction phases of every facial expression and EMG channel.

### Topographical heat maps for visualization of the EMG activity

To illustrate the topographic distribution of the EMG activity over the face for both schemes, topographical heat maps were calculated as in the previous study^16^. Briefly, a modified 4-nearest neighbor interpolation of the EMG RMS values with the inverse square of the distance as weight was used to produce the heat maps^21,22^. As the conducting area of the face is not spherical, the weight of the most distant (fourth) electrode was steadily pulled to zero in the vicinity of the change between two fourth neighbors^23^.

### Statistics

All statistical analyses were performed using IBM SPSS Statistics 25 (Chicago, IL). The results are presented as means ± standard deviation (SD) of the study sample in the same way as recently reported^17,18^. A linear mixed-effects model (LMM) was applied separately for each scheme to evaluate the main effects of the parameters “emotion,” “side,” and “electrode position,” together with their interactions. “side” means the body side. “electrode position” means the 11 muscles positions in case of the Fridlund scheme and the 8 topographical positions of the Kuramoto scheme. “emotion”, “side”, and “electrode position” were modeled as fixed effects with a random intercept per subject. First, all main effects and interactions were calculated, but for the final analysis, only the significant main effects together with significant interactions remained in the calculation. Adjustment for multiple comparisons for differences between the tested facial movements was performed by the least significant difference. The significance level was set to 5%. Additionally, the LMM was also applied to the upper and lower face for possible regional effects. To allow a comparison to other data sets, the dimensionless coefficient of variation (CV) was calculated additionally. The CV was calculated as the ratio of the standard deviation (SD) to the mean as a percentage ([CV = standard deviation/mean] × 100) for each individual and the different settings. The lower the CV, the more precise was the estimate. Thus, when SD and CV correspond in showing high values, the therewith-identified varying activity maxima should be considered in the evaluation of the amplitude distribution patterns.

The four standard methods were used to test the re-test reliability: Intraclass correlation coefficient (ICC), standard error of the measurement (SEm), standard error of the mean (SEM), and coefficient of variation of method error (CVME statistics. Data are presented as mean ± 95% confidence intervals (CI). Comparisons were performed to test the reliability of the four trials in each session (intrasession reliability), between the two sessions at the same day (within day reliability) and between the two days (between day reliability). Intraclass correlation coefficient (ICC) statistics expressed with lower and upper borders (i.e. 95% CI, also applies for the other reliability parameters) were used to analyze the retest reliability of the normalized EMG amplitudes. The higher the ICC value, the more precise is the estimate. ICC values less than 0.5 are indicative of poor reliability, values between 0.5 and 0.75 indicate moderate reliability, values between 0.75 and 0.9 indicate good reliability, and values greater than 0.90 indicate excellent reliability^24^. The SEm statistics allow a calculation of the standard deviation (SD) of mean values (MV) at (theoretically) infinite repetition of measurements per subject over group using the formula: SEm = SD * √(1-ICC). The SEM calculates the- SD of distribution of deviations in repeated estimation of values of a population: SEM = SD/√(N). SEM can be used to define the uncertainty range of a measurement or the minimum treatment effect (± SD, i.e. the 68% certainty that there is no random effect when leaving the range). Finally, the CVME allows a relative description of the SEM in percent: CVME = 2 x (SEM/√(2))/sum(MV1 + MV2)).

### Ethics statement

Written informed consent was obtained from all participants. The ethics committee of the Jena University Hospital approved the study (No. 2019-1539).

## Results

### Topography of the high-resolution facial surface electromyography (HR-sEMG) activation during emotional expressions

**Figure 2:**
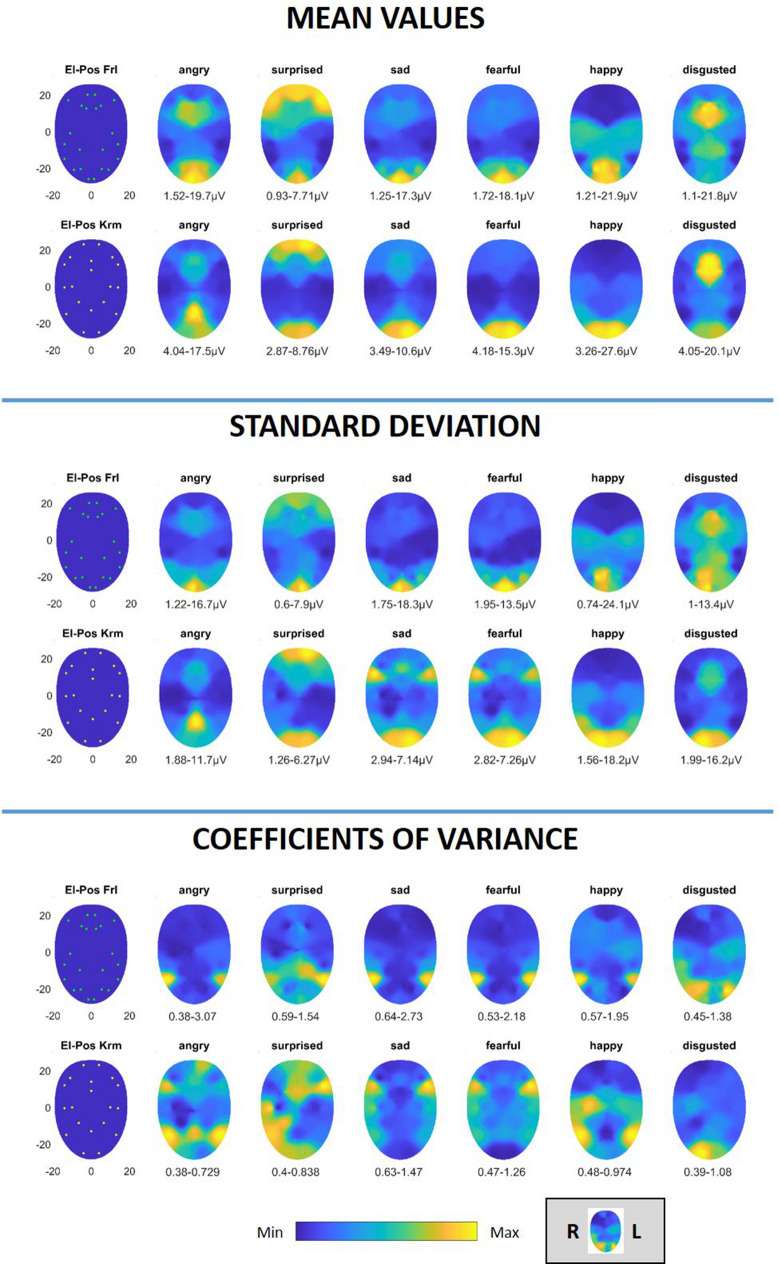
Activation heat maps of facial muscle activity during the imitation of the six basic emotions using the Fridlund scheme (El-Pos Fri) and the Kuramoto scheme (El-Pos Krm). According to the provided color bars, blue stands for low values and yellow for high values. Below each map, the respective minimal and maximal values are provided. Top: Mean values; Center: Standard deviation; Bottom: coefficient of variance. The right side of the face is always shown on the left side of each heat map. The left side of the face is shown on the right side of each heat map.

The heat map presentation of the mean values of muscle activity of the six basic emotions is shown in Fig. 2 top. Activation characteristics showed systematic regional distribution patterns. On visual inspection, both the Fridlund and the Kuramoto scheme showed similar patterns. The muscle activation patterns of the Kuramoto scheme were less variable in muscles reaching the midline. The variability of these values across the face is provided by the presentation of the SD in Fig. 2 center and in an amplitude-normalized fashion, i.e., CVs, and are presented in Fig. 2 bottom. Some facial expressions showed a very low inter-individual variability of the activation patterns (more blue color in Fig. 2, for instance, “sad” or “fearful”), whereas others showed a higher variability (more yellow color in Fig. 2, for instance, “surprise” or “disgusted”). Overall, the variability of the activation in μV was very low. SD and CVs were slightly lower for the Kuramoto scheme. Only for “disgusted”, SD values were lower for the Fridlund scheme.

**Figure 3:**
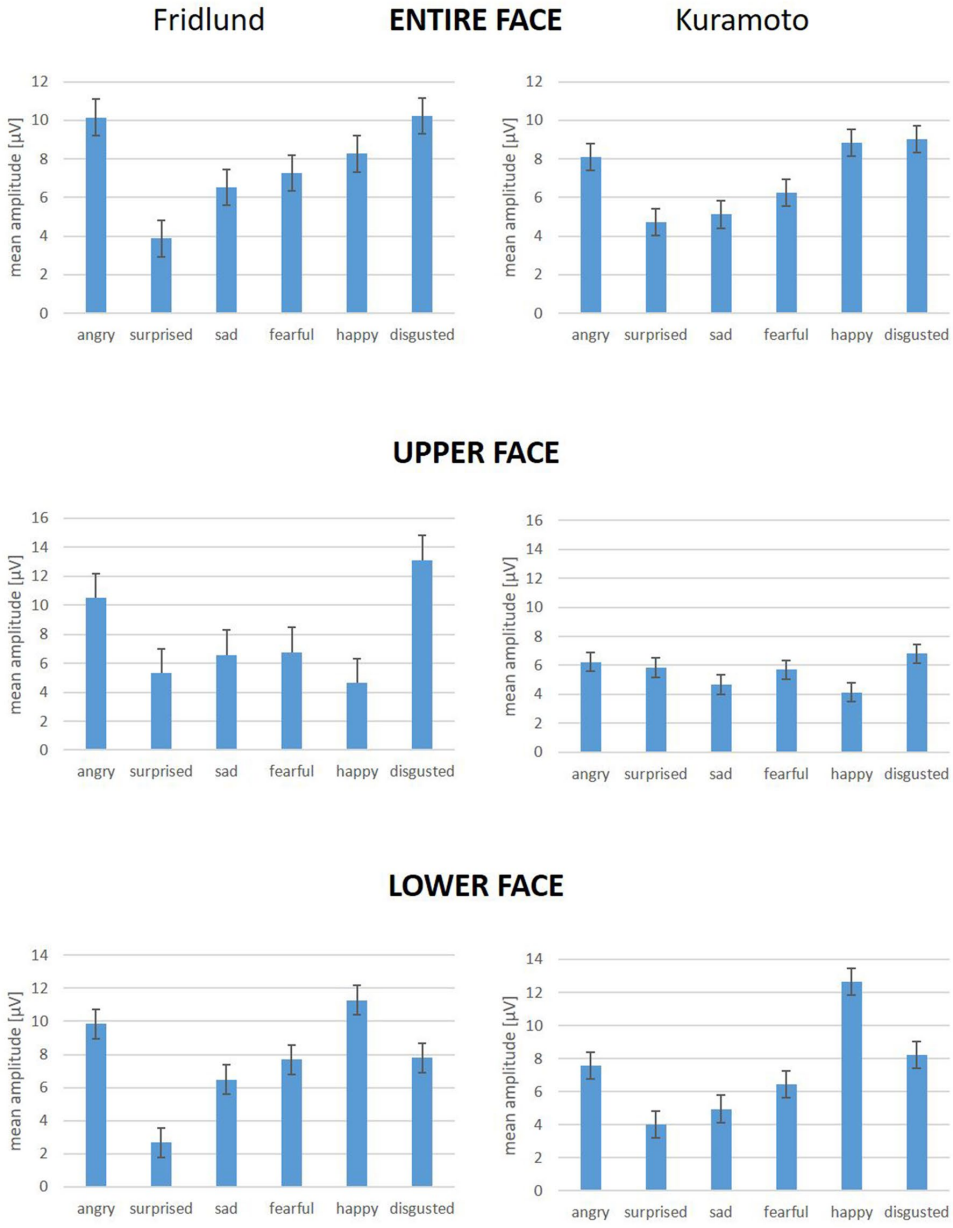
Mean RMS amplitudes (± 95% confidence intervals) of facial muscle EMG recordings in all participants during the imitation of the six basic emotions for the Fridlund scheme (left side) and the Kuramoto scheme (right side): Top: for the entire face; Center: for the upper face; Bottom: for the lower face. The face was divided through a horizontal line in height of the infraorbital margins into an upper and lower face.

Figure 3 displays the summarized muscle activity over all EMG electrodes for the entire facial face (Fig. 3 top), for the upper face (Fig. 3 center) and for the lower face (Fig. 3 bottom). “Angry” and “disgusted” showed the highest muscle activity. “Surprised” and “sad” the lowest activity. Overall, the activity was higher in the upper compared to the lower face. Much more activity within the upper face compared to the lower was seen for “disgusted”. Vice versa, more activity was measured in the lower face than in the upper face for “happy”. There were differences between the Fridlund scheme and the Kuramoto scheme: Whereas “sad” and “fearful” showed more activity in the Fridlund scheme for the facial muscles of the lower face, it was vice versa for the Kuramoto scheme.

### Using high-resolution facial EMG activation for the differentiation between the six emotional expressions

In Table 1, the results of the LMM calculations of the pairwise comparisons between all six emotional expressions are provided for the RMS and normalized datasets. The sEMG recording allowed highly significant discrimination between most of the emotional expressions (p < 0.001) but a few failed (p > 0.05). When the recordings of the entire face were included, the recordings using the Kuramoto scheme could differentiate between all six emotions with very high reliability with one exception: The differentiation between “happy” and “disgusted” was not significant. Using the Fridlund scheme was also very reliable when using data from the entire face, with two exceptions: “sad” versus “fearful” and “angry” versus “disgusted” could not significantly be differentiated. If only recordings of the lower face were used for the calculations, the discrimination was better for both schemes only with one exception: “fearful” and “disgusted” could not be significantly differentiated with the Fridlund scheme. All six emotions could be differentiated with high precision when using the data for the lower face and the Kuramoto setting. When only data of the muscles of the upper face were used, the Fridlund scheme was much less reliable. Here, the Kuramoto scheme performed much better. Only “surprised” and “fearful” could not be significantly separated when using only data of the upper face.

**Table 1:** Results of the in-between comparisons of the six basic emotion by applying a linear mixed-effects model.

Fridlund						Kuramoto					
p-values	Surprised	Sad	Fearful	Happy	Disgusted	p-values	Surprised	Sad	fearful	Happy	Disgusted
**Entire face**											
Angry	< 0.001	< 0.001	< 0.001	< 0.001	n.s	Angry	< 0.001	< 0.001	< 0.001	< 0.001	< 0.001
Surprised		< 0.001	< 0.001	< 0.001	< 0.001	Surprised		< 0.001	< 0.001	< 0.001	< 0.001
Sad			n.s	< 0.001	< 0.001	Sad			< 0.001	< 0.001	< 0.001
Fearful				< 0.01	< 0.001	Fearful				< 0.001	< 0.001
Happy					< 0.001	Happy					n.s
**Upper face**											
Angry	< 0.001	< 0.001	< 0.001	< 0.001	< 0.001	Angry	< 0.005	< 0.001	< 0.001	< 0.001	< 0.001
Surprised		n.s	n.s	n.s	< 0.001	Surprised		< 0.001	n.s	< 0.001	< 0.001
Sad			n.s	< 0.05	< 0.001	Sad			< 0.001	< 0.001	< 0.001
Fearful				< 0.005	< 0.001	Fearful				< 0.001	< 0.001
Happy					< 0.001	Happy					< 0.001
**Lower face**											
Angry	< 0.001	< 0.001	< 0.001	< 0.001	< 0.001	Angry	< 0.001	< 0.001	< 0.001	< 0.001	< 0.001
Surprised		< 0.001	< 0.001	< 0.001	< 0.001	Surprised		< 0.001	< 0.001	< 0.001	< 0.001
Sad			< 0.001	< 0.001	< 0.001	Sad			< 0.001	< 0.001	< 0.001
Fearful				< 0.01	n.s	Fearful				< 0.001	< 0.001
Happy					< 0.001	Happy					< 0.001

Fridlund scheme on the left side, and Kuramoto scheme on the right side, for the entire face (top), upper face (center), and lower face (bottom). P-values are presented. The border between the upper and lower half of the face was defined through a horizontal line along the infraorbital margin.

### Re-test reliability of high resolution facial electromyography for characterization of the six basic emotions

The complete set of calculations is presented in the Supplementary information for the Fridlund scheme and for the Kuramoto scheme (Supplementary Fig. S1). ICC; SEm, SEM, and CVME were calculated for both the Fridlund and the Kuramoto scheme. Regarding the Fridlund scheme, intra-session ICC was highest for “happy” (0.950) and lowest for “fearful” (0.796). The inter-session ICC did not differ relevantly between the four sessions but were lower for the between day comparisons (within day: 0.727–0.857, between day: 0.581–0.688). The intra-session SEm was highest for “fearful” (1.465) and lowest for “happy” (0.527). The inter-session SEm was not much different for all emotions (within day: 0.492–1.276, between day: 0.818–2.619), with “surprised” showing lowest SEm values. The intra-session SEM (0.272–0.654) and inter-session (within day: 0.192–0.477, between day: 0.252–0.696) was again lowest for “surprised”. The intra-session and the inter-session CVME did not show relevant differences (intra-session: 7.53–10.38%, within day: 5.17–6.87%, between day: 7.36–8.53%).

Regarding the Kuramoto scheme, intra-session ICC was highest for “happy” and lowest for “fearful” like for the Fridlund scheme (0.761–0.942). The inter-session ICC showed more variability between the four sessions (within day: 0.68–0.834, between day: 0.475–0.756). The intra-session SEm was highest for “fearful” and “disgusted” and lowest for “surprised” and “sad” (0.599–1.477). The inter-session SEm was not much different for all emotions except for “happy” and “disgusted” with higher SEm values (within day: 0.609–0.890, between day: 1.087–2.257). The intra-session SEM and inter-session was lowest for “surprised” like is was seen for the Fridlund scheme (0.300–0.607). The intra-session and the inter-session CVME did not show relevant differences (intra-session: 5.44–8.48%, within day: 3.88–5.51%, between day: 5.48–7.33%). Overall, the Kuramoto scheme produced better estimates than the Fridlund scheme.

## Discussion

Ekman and Friesen discussed in 1976 the question if observers of a psychophysical experiment can make accurate inferences about different facial movements only through visual observation^10^. They stated already that electromyography (EMG) of the facial muscles has the advantage not to be focused upon what is visible in the face. Instead, EMG could measure visible but also non-visible changes in facial muscle tone. Actually in these days they thought that “it is unlikely that surface electrodes could distinguish the variety of visible movements which most other methods delineate”^10^. Meanwhile, especially studies using HR-sEMG have shown sEMG is a very reliable method to differentiate between different functional expressions in the face (eye closure, pursing lip, et cetera) of healthy probands and patients with facial motor diseases^14,17,18^.

Beyond functional expressions, facial expressions might be even more important in humans to display personal emotions and indicate an individual’s intentions within a social situation and, hence, are extremely important for social interaction. From a scientific point of view, this is not only important in psychological experiments for psychophysiological measures. This is also important for clinical settings as patients with impaired mimic function like, for instance, patients with facial palsy or Parkinson’s disease also experience impaired social interactions^25,26^. Beyond eye protection, support of eating, drinking, and speaking, facial muscle movements are indispensable signals to express emotional states^27^. Ekman has developed a categorical emotion model suggesting a set of basic emotions^6^. The standard system to measure emotions is the Facial Action Coding System (FACS)^9^. FACS visually assesses facial expressions identifying discrete facial muscle movements called Action Units (AUs) without use of EMG by FACS trained observes or nowadays also by automated image analysis^28^. According to the FACS approach, each AU codes the fundamental actions of individual or groups of muscles typically seen while producing facial expressions of emotion^29^. There is, however, an ongoing debate as to whether reading from the facial surface can suffice for detection of distinct emotional states^3,11^. Furthermore, FACS studies classifying the six basic emotions show variable AU expression patterns^30^. The reason might be that similar configurations of facial movements variably express instances of more than one emotion category^1^. When performing automated facial coding and EMG of the zygomaticus and the corrugator muscle synchronously, unpleasant pictures were classified by an automated facial coding variably as neutral or unpleasant stimuli, whereas this was highly reliably distinguishable with EMG^5^.

The present study is the first to show that HR-sEMG is highly reliable to distinguish between all six basic emotions when posed by healthy adult probands. The key was the use of multi-channel recordings. In 1986 Fridlund and Cacioppo published the citation classic standardizing the use of facial sEMG for psychophysical experiments^16^. They recommended a sEMG recording of ten facial muscles (and one chewing muscle) to have a realistic setting. Although frequently cited when using sEMG applications for confirmation in experiments analyzing emotions, often only selected muscles are recorded: for instance, the zygomatic major muscle and the orbicularis oculi muscle for a positive affect (“happy” emotion) and the corrugator supercilii muscle as negative control (“sad” emotion”)^31–33^. This does not allow a reliable discrimination for posed “happy”, “sad”, and “angry” emotions^34^. Especially the valence assigned to the zygomatic muscle is very variable^35^. The present study shows that the activation during the six basic emotions is much more complex involving many if not all facial muscles.

More important, by applying HR-sEMG, the activity of facial muscles in the lower face seem to be the better discriminator than muscles in the upper face. This is a very important finding as it contradicts the paradigm to focus EMG studies on the upper face in psychological experiments^31–33^. Especially for the differentiation of emotional expressions, EMG analysis of the lower face is very important^36^. Another disadvantage of the focus only on the corrugator supercilii and zygomatic major muscles: If the other muscles are not included into the analyses, they might only “disturb” the answers in the corrugator supercilii and zygomatic major muscles as crosstalk making the analysis difficult^36^. One should be aware that such a limitation (in the upper and/or lower face) to only one to three facial muscles limits the meaningfulness of studies analyzing the validity of automated emotion classification^2^.

In clinical, but also experimental settings, patients or participants are examined several times. It might be that the stimuli vary or that the measurements are performed at different days before and after an intervention^18^. Hence, it is very important that the used sEMG scheme can be applied in such a way that a variability of the EMG recordings is limited as much as possible^18^. EMG schemes like the Fridlund scheme are dependent on a repeated exact positioning on the related facial muscles. The repeated measurements are influenced by the inter-electrode distances, crosstalk, and the influence of both on the sEMG recordings. Furthermore, recording of fewer muscles can lead to insufficient test–retest reliability^32^. Overall, the Kuramoto scheme showed the better re-test reliability values. Furthermore, the EMG muscle activity values showed less inter-individual variability for the Kuramoto scheme, and the discrimination between the six basic emotions was better, especially in the upper face than for the Fridlund scheme. We expected this result, because the Kuramoto scheme is not dependent on exact positioning on anatomically defined facial muscles. We reported this already regarding facial functional movements^18^. For the emotional movement patterns in the presented study, the re-test statistics were not relevantly different between both schemes and in-between the six emotions. Overall, the Kuramoto scheme can be recommended for its better discriminatory function.

The present study has limitations. Most work on emotional expressions relies on posed facial behavior, often depicted in a static position^37^. The posed displays might be of idealized nature. Spontaneously displayed expressions can be more complex and can have an increased ambiguity of their emotional content^38,39^. To use HR-sEMG in the presented form for settings with spontaneously displayed expressions seems not to be feasible, because the electrodes and wiring would be too disturbing. A wireless solution would be needed, but current wireless applications do not yet allow to cover the complete face or are limited to the upper face^3,40^.

## Conclusions

High-resolution sEMG recordings of healthy probands showed that the entire facial muscles and not only selected and anatomically defined facial muscles were involved in the expression of the six basic emotions. The Kuramoto scheme performed better for the discrimination of the basic emotions, especially when only data of the lower face muscles were used. Both the Fridlund and the Kuramoto scheme show a high intra-session and inter-session test–retest reliability.

### Supplementary Information

Supplementary Legends.

Supplementary Figure S1.

## Data Availability

The original contributions presented in the study are included in the article and in the supplementary material. Further inquiries can be directed to the corresponding author.
