# Supplementary material for: High-resolution surface electromyographic activities of facial muscles during the six basic emotional expressions in healthy adults: a prospective observational study
Source: Sci Rep. 2023 Nov 6;13:19214. doi: 10.1038/s41598-023-45779-9 (PMC10628297; doi:10.1038/s41598-023-45779-9)
Supplement: Supplementary file 1 — Supplementary Legends. [file 41598_2023_45779_MOESM1_ESM.docx]

**Supplementary Figure S1.** Re-test reliability for the six emotions for the Fridlund (F) and the Kuramoto (K) scheme using intraclass correlation coefficient (ICC), standard error of measurement (SEm), standard error of mean (SEM), and coefficient of variation of method error (CVME) calculations. Data presented as mean ± 95% confidence intervals (CI). Compared were the intra-session reliability (light and dark blue), the within day reliability (light and dark orange), and the between day reliability (light and dark green).
