# Supplementary figures and images for: High-resolution surface electromyographic activities of facial muscles during the six basic emotional expressions in healthy adults: a prospective observational study
Source: Sci Rep. 2023 Nov 6;13:19214. doi: 10.1038/s41598-023-45779-9 (PMC10628297; doi:10.1038/s41598-023-45779-9)

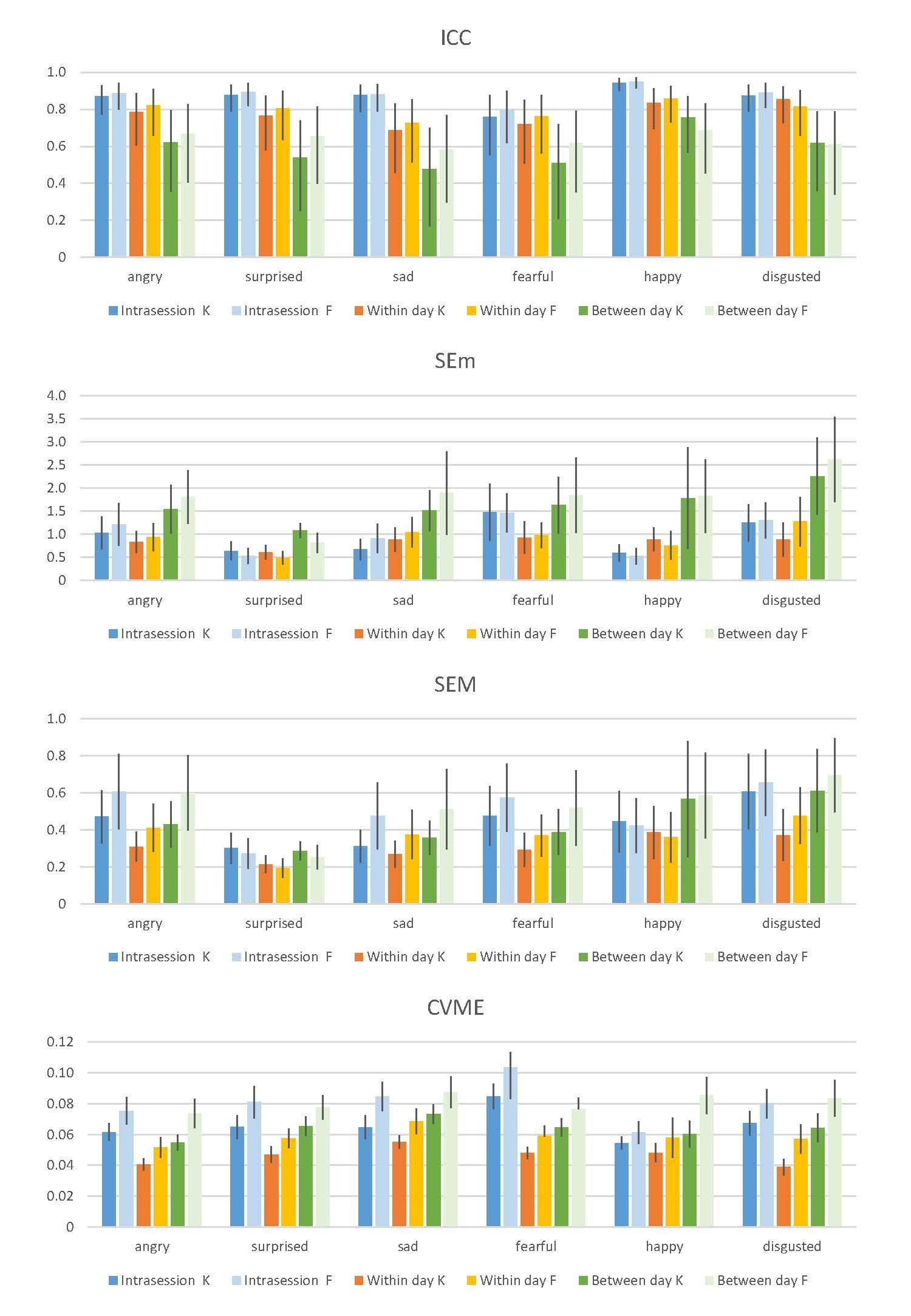

Supplement: Supplementary file 2 — Supplementary Figure S1. [file 41598_2023_45779_MOESM2_ESM.jpg]
